# Integration of multiple geospatial applications and intelligence for responding to COVID-19 in Ghana

**DOI:** 10.4314/gmj.v55i2s.3

**Published:** 2021-06

**Authors:** Alex B Owusu, Seth K Afagbedzi, Delia A Bandoh, Joseph A Frimpong, Isaac N Kissiedu, Ben E Aikins, Richmond Hinneh, Ernest Kenu

**Affiliations:** 1 Department of Geography and Resource Development, University of Ghana, Legon, Accra; 2 Department of Biostatistics, School of Public Health, University of Ghana, Legon, Accra, Ghana; 3 GFELTP, Department of Epidemiology and Disease Control, School of Public Health, University of Ghana, Legon, Accra, Ghana

**Keywords:** ArcGIS, geospatial, COVID-19, pandemic, distribution

## Abstract

**Objective:**

We describe the use of integrated geospatial applications for the provision of access to timely and accurate data on samples, visualisation of Spatio-temporal patterns of cases and effective communication between field sample collectors, testing laboratories, Regional Health directors and Government Decision Makers.

**Design:**

This study describes how an integrated geospatial platform based on case location and intelligence was developed and used for effective COVID-19 response during the initial stages of COVID-19 in Ghana.

**Data Source:**

Collector for ArcGIS, ArcGIS Survey123

**Main outcome measure:**

successful development and deployment of integrated geospatial applications and analytics.

**Results:**

The Collector for ArcGIS app was customised to collect COVID-19 positive cases location information. Survey 123 was introduced as a COVID-19 contact tracing application to digitise the case-based forms and provide real-time results from the laboratories to GHS and other stakeholders. The laboratory backend allowed the testing laboratories access to specific information about each patient (sample) collected by the fieldworkers. The regional supervisors' backend web application provided accessing test results for confidentiality and timely communication of results.

**Conclusion:**

Geospatial platforms were successfully established in Ghana to provide timely results to Regional Health Directors and Government decision-makers. This helped to improve the timeliness of response and contact tracing at the district level.

**Funding:**

The development and deployment of the application, COVID-19 pandemic response and writing workshop by the Ghana Field Epidemiology and Laboratory Training Programme (GFELTP) was supported with funding from President Malaria Initiative – CDC, and Korea International Cooperation Agency (on CDC CoAg 6NU2GGH001876) through AFENET and the United States Agency for International Development (USAID) through Results for Development (R4D).

## Introduction

Coronavirus disease 2019 (COVID-19), also known as severe acute respiratory syndrome coronavirus 2 (SARS-CoV-2) was first detected as an outbreak of respiratory illness in Wuhan City, Hubei Province, China.^1^ China was the first country to report to the World Health Organization (WHO) on December 31, 2019 that pneumonia of unknown cause. Subsequently, Thailand, and Japan notified WHO of confirmed cases of novel coronavirus known as 2019-nCoV.^2^ The WHO declared the COVID-19 outbreak a public health emergency of international concern on January 30, 2020.^3^,^4^

The WHO, on March 11, declared COVID -19 a pandemic, and since then, the virus has spread to all six continents and 213 countries.^5^ The virus has infected more than 23 million people globally, with a death toll of more than 800,000 as of August 2020.^6^ The world has previously experienced other coronaviruses such as Severe Acute Respiratory Syndrome (SARS) reported in China in 2003 and the Middle East Respiratory Virus (MERS CoV) reported in Saudi Arabia in 2012.^7^

At the initial stages of the pandemic, countries adopted various response strategies, including geospatial approaches to prevent the further spread of the virus and mitigate its effects.^8^ China's first phase of public health response to COVID-19 focused on short-term measures to stop the virus from spreading from Hubei to the rest of the country and included “extreme lockdowns” and regional quarantines.^9^

China, among other strategies, has implemented a differentiated, location-specific response to prevent the spread so that public health measures are tailored to the differing realities on the ground. For example, measures adopted in Wuhan were very different from those applied in other places such as Chengdu or Shanghai.^10^

In Ghana, the first two coronavirus cases were reported on March 12^th^, 2020, and as of 30^th^ March 2020, the total confirmed COVID-19 cases were 152, with five deaths.^11^ Majority of the cases were concentrated in Accra and Kumasi metropolitan areas, the two most populated cities of the country. On March 30^th^ 2020, the government of Ghana imposed a partial lockdown of Accra, Kasoa and Kumasi as additional measures to those adopted two weeks earlier, which included a ban on all public gatherings, closure of schools, churches, mosques, other places of worship and a mandatory quarantine of all travellers that arrived in the country 48hrs before the closure of the country's borders.^12^,^13^ Announcing the lockdown, the President indicated that his government had five (5) key objectives to combat the pandemic in Ghana, and these were to; 1. Limit and stop the spread of the virus; 2. Contain the spread of the virus; 3. Provide adequate care for the sick; 4. Limit the impact of the virus on social and economic life; 5. Inspire the expansion of the domestic capability and deepen self-reliance. All these measures were aimed at limiting the spread of the virus.

Since the detection of Ghana's first case on March 12th, 2020, several strategies have been adopted by the health authorities to control the epidemic. Ghana Health Service (GHS), the Ministry of Health (MoH) 's implementing body, was tasked with establishing effective mechanisms for disease surveillance, prevention, and control and leading the COVID-19 response.^14^ One key strategy of GHS response is the “3 Ts approach” - Testing, Tracing, and Treatment and provision of data from this strategy in real-time. GHS, therefore, rolled out the “SORMAS” (Surveillance Outbreak Response Management & Analysis System) application as a national electronic real-time platform for surveillance and outbreak response.^15^ An earlier assessment of SORMAS to determine its readiness to be used as a response tool for the COVID-19 outbreak in Ghana revealed some challenges, including limited coverage across the country and some in-depth geospatial analysis, which would be relevant for key response strategies. As the outbreak progressed, there was a need to expand the geospatial capacities of the SORMAS platform and acquire additional logistics to expand its reach across the country.

As a stopgap measure, the School of Public Health (SPH), Ghana Field Epidemiology and Laboratory Training Program (FELTP), and the Department of Geography and Resource Development at the University of Ghana developed a system to complement the existing structures as the challenges identified with the rollout of SORMAS were being addressed. These measures were to help provide geospatial data on cases and provide nationwide coverage in the meantime. Consequently, a geospatial mobile application was developed to demonstrate the power of integrating multiple geospatial applications with spatial intelligence capabilities in effectively managing the COVID-19 situation in Ghana.

This paper demonstrates the use of an integrated geospatial application and analytics via cloud system for the provision of access to timely and accurate data on samples, visualisation of the Spatio-temporal pattern of the case and effective communication between field sample collectors, testing laboratories, Regional Health directors and Government Decision Makers.

## Methods

### The reference point for application

**Figure 1:**
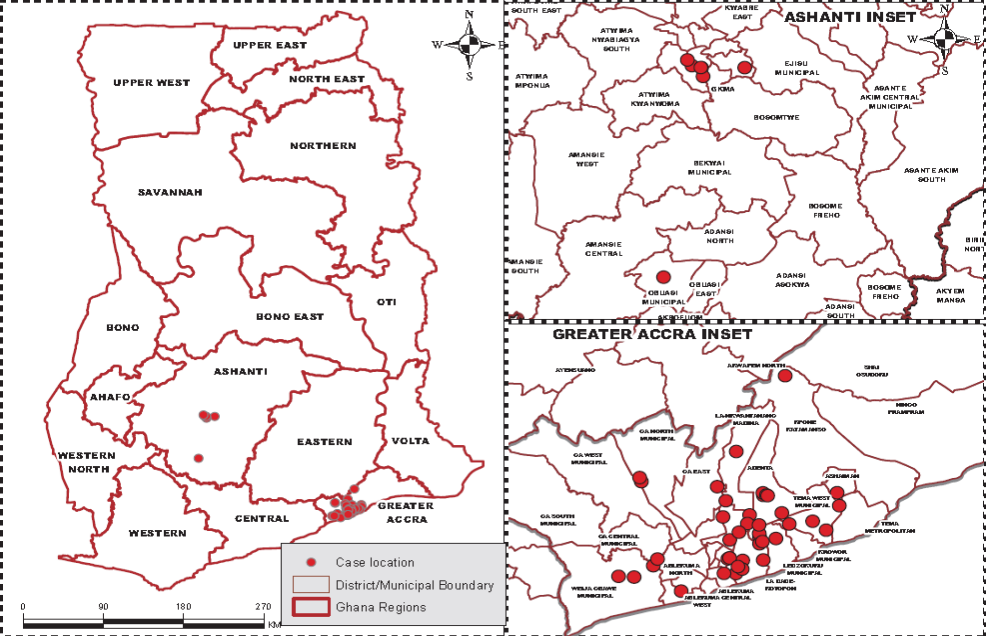
Map of Ghana with Ashanti and Greater Accra Regions Inset and location of COVID 19 cases as of 31^st^ March 2020

The application was initially developed based on gaps identified in the response at the two epicentres for the pandemic in Ghana, that is, Greater Accra Metropolitan Area (GAMA) and Greater Kumasi Metropolitan Area (GKMA), hence referred to as COVID-19 hotspots (Figure 1).

### Design

The application was designed by integrating a geospatial platform based on case location and intelligence using ArcGIS and Survey 123.

### Description of the platform

The platform provided an opportunity to integrate four different but complementary applications for contact tracing activities, receiving, testing, reporting test results, visualisation of sample collection patterns and Spatiotemporal patterns of cases. The platform had unique functional parts which included: Geospatial mobile app for the data collection on the field; Real-time spatial distribution of cases; Portal for labs to update test results; Portal for district supervisors to monitor work of field staff; Portal for regional directors and district directors to visualise the distribution of the cases and download line list and Barcode scanner for sample labelling and tracking. There was also an interactive dashboard in the office of the President of the Republic of Ghana, which enabled the Presidency (WAR ROOM) to have real-time access to what was happening in the field.

These different but complementary applications were:

### Collector for ArcGIs

The Collector for ArcGIS developed by Environmental Systems Research Institute (ESRI) is a Geographic Information System (GIS) enabled mobile application that works on Android and iOS Operating Systems and can collect the location and attribute data about any phenomenon of interest.^16^ In this case, the app was customised to collect COVID-19 positive cases location information. ArcGIS Online served as the data storage repository whenever data was submitted from the Collector app, making it possible to get the GIS data in real-time for visualisation and analysis like heat maps and risk zones. The ArcGIS Collector uses a spatial layer with attribute information either developed in ArcMap, ArcGIS Pro, or even online. The Spatial layer enables users to see the location or point of interest, whereas the attribute information usually displays all sorts of information collected from the field.

### ArcGIS Survey 123

The ArcGIS Survey 123, just like the Collector for ArcGIS, was a data collection system developed by ESRI to facilitate field data collection in the form of a digital questionnaire to collect location information.^17^ The survey 123 supports Android, iOS, and Windows operating systems. The survey 123 platform enables users to customise digital forms or questionnaires to match a paper one.

Survey 123 was introduced as a COVID-19 contact tracing application to digitise the case-based forms and provide real-time results from the laboratories to GHS and other stakeholders. The primary users were the field workers and health facilities. The field workers included contact tracers and Community Surveillance, Team. The application was installed on mobile devices, which in this situation was a tablet – Samsung Tablet 10.8 – with an Android operation system. Each team logged into this mobile application on a tablet and connected to the customised ArcGIS Survey 123 for the ArcGIS platform. The backend database contained the case-based investigation form, designed and approved for use by the GHS.

### Laboratory Backend Web Application

The laboratory backend was a secured web portal capable of providing real-time access to the laboratory data.^18^ The application allowed the testing laboratories access to specific information about each patient (sample) collected by the fieldworkers. The labs received samples from the field with tagged barcodes. With the help of barcode scanners, samples were scanned to retrieve their information in the database. The information was then updated, marked as received, and test results updated to ensure a seamless flow of samples from the field to the laboratories. It had a visualisation map that showed the locations where the samples were collected and their test results status.

### Regional Supervisors backend web applications

This application has a similar structure and functionalities as the lab, except it is read-only and downloadable. It was mainly used for accessing test results for confidentiality and timely communication of results. Regional Directors of Health and Regional Supervisors of the COVID-19 program, appointed by Ghana Health Services, were the primary target. Hence, 16 dedicated web portals were created for regional directors and supervisors to access and download the lab test results of their respective regions.

### Interactive Dashboard in the office of the President

The ArcGIS Dashboard is a visualising platform that can display diverse infographics, including graphs, charts, and values depending on the nature of data, and the result demanded from visuals.^19^ This system component is connected to the data feed from the ArcGIS collector and ArcGIS Survey 123 for contact tracing applications to the President's war room. Data was updated immediately after information was uploaded on the field. The Dashboard displayed the various recorded cases in the form of points representing their locations. To simplify the information on the Dashboard for decision-makers to see what was happening, various sections were created to display different forms of infographics.

### The structure of the related tables of the application's database

**Figure 2:**
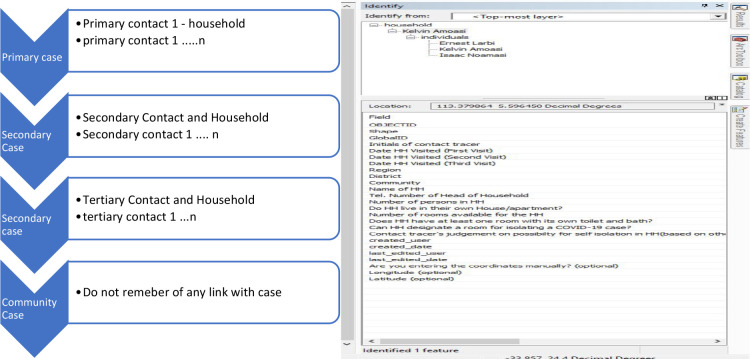
Contact Tracing and Community Case Tracking Relational Tables

The preparation of the spatial and attribute layer was done in ArcGIS Pro. In ArcGIS Pro, a database was created for households. Point features were used to represent households. To generate a form of decision tree to determine the first case in a household and its contacts and household indication, a relationship was built as part of the database to link household members to the first case who would then serve as contacts. The back end of the applications was the related table showing sample and related samples (see Figure 2). Selecting a sample from the map shows its related tables. Figure 2 shows how individuals are mapped or sampled, and the design of the related tables for contact tracing enhanced contact tracing and community surveillance.

On the household layer, a ‘GlobalID’ is generated by default for every single record within this layer. The attribute information to be collected field workers on the household included; the date that the household was visited, region, district, community, name of household (Household owner/head name), telephone number of the house owner, number of persons in the household, number of rooms available in the household. On the other hand, an attribute table named ‘individuals’ was created to store the information on members of the household. Information to be collected by the field workers included the names of individuals in the household, age of the individual, the gender, telephone number, travel status, health condition, whether or not a sample was taken in that house, the date that the sample was taken, laboratory results if available. These parameters appeared on the Collector for ArcGIS as a form for field workers to fill. To ensure data quality, some of these questions were made mandatory fields for sample collectors. Failure to populate these mandatory fields would not enable data to be submitted from the field. For the spatial layer and the attribute table to communicate, such that when a user clicks on a point, all other members related to that household appears, a foreign key which in this case was the Global ID of the household, was introduced in the relationship enabling the spatial data to link up with a piece of corresponding attribute information. A one-to-many relationship was enabled, making it possible for more than one individual to be linked to a household. The Geodatabase, which contains the household layer, the individual attribute table and the relationship class, was then published to ArcGIS Online. A web map was created in ArcGIS online to enable the field team to access the created layers on the collector for ArcGIS. With Android tablets, sample collectors were able to access these layers on the field. The accuracy of locations was set to 10m so that the system would not accept anything above 10m.

### Ethical consideration

Approval for the study was obtained from Ghana Health Service Ethics Review Committee (GHS-ERC 006/05/20). The confidentiality of data was maintained throughout the study. The data collected is only available to the administrator of the database. Once the data has been synced, the field data collectors don't have access. The data is anonymised before it is shared. Data was kept on password-protected computers and was only accessed by authorised persons.

## Results

### Geospatial Applications for COVID-19 Data Collection

**Figure 3:**
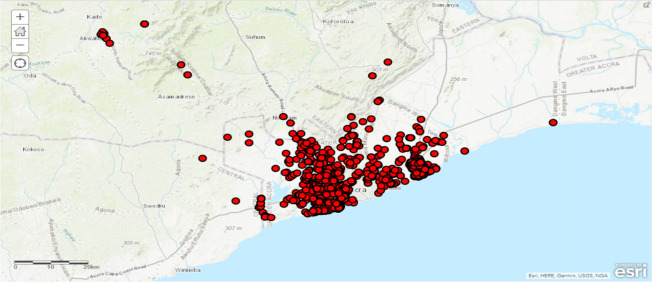
Mapped cases from the ArcGIS collector

The Geospatial mobile app for field data collection- Figure 3 shows ArcGIS Collector and Survey 123 applications electronically collected information contained in the case-based forms developed by GHS for COVID-19 data collection. In addition to this, GPS locations were collected for each sample. The applications also collected information on suspected and potential cases. This information was sent to the laboratory backend for updating of tests results. Figure 3 shows the interface of the geospatial contact tracing a mobile application with the Electronic Case-based Form

The electronic case-based investigation form was categorised into five main sub-sections, with each section containing several questions. It started with survey information, followed by patient personal information, then the clinical information, exposure and travel information over the last 14 days before the survey and information on sample collection. The fieldworkers had the chance to edit and correct errors within time before the physical sample got to the labs.

Figure 3 shows case locations mapped immediately on ArcGIS Online, ready for analysis and interpretation after the fieldworkers submitted their data.

**Figure 4:**
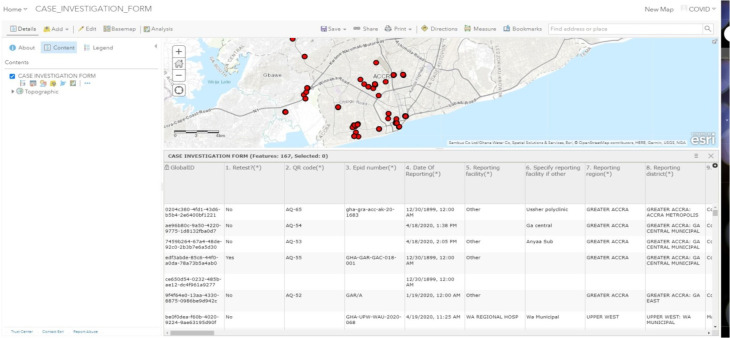
Mapped sample point and attributes from ArcGIS Survey 123

Figure 4 shows sample points and attributes from ArcGIS Survey 123. Immediately the field staff submit information entered into the electronic CASE_INVESTIGATION_FORM. The laboratories receive it in real-time. The fieldworker could crosscheck data submitted against the number of samples, and field staff could count the entries of all sent data as shown below. Necessary corrections could also be made if any, and the data re-submitted.

### Laboratory Backend Web Application

**Figure 6:**
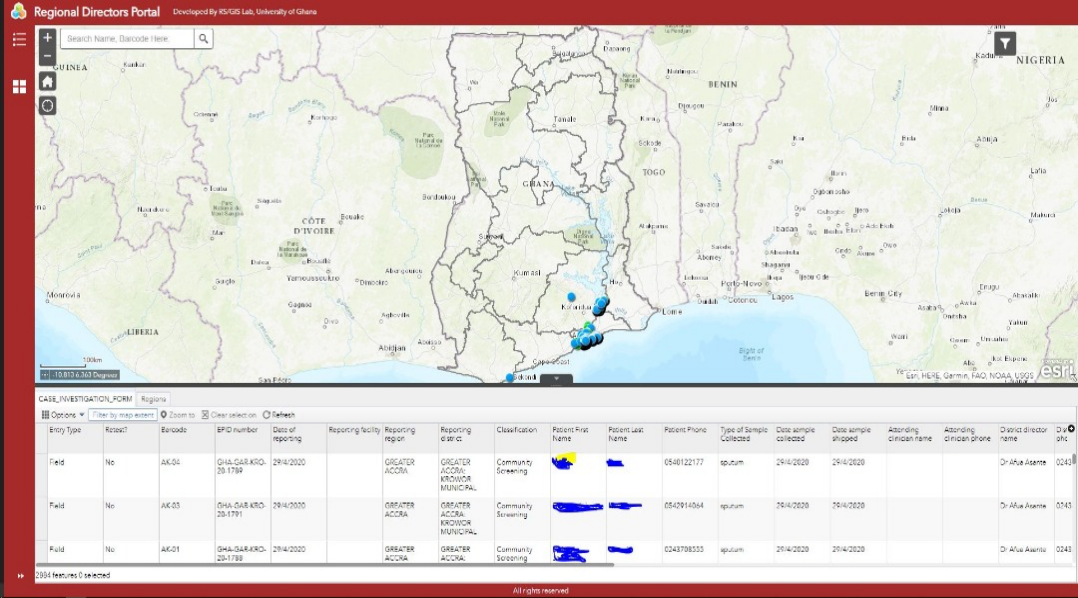
Regional directors' and supervisors' portal

Figure 6 shows the user interface of the lab backend web application. The labs receive samples from the field with barcodes labelled on the samples. Barcode reading scanners were used to scan the barcodes on the sample. This query field sample data in the database and mark as received and update test results. The application has a visualisation map and a meaningful display of the locations where the samples were collected and their test results status. The sample points that appeared red were results that tested positive for COVID-19, and the green coloured sample points were negative. Sample points pending confirmation of test results were coloured in blue whilst invalid sample points were coloured in black.

Figure 6 shows the regional directors and supervisors' portal and data. This interface provides the line list of individuals whose samples were taken, including the personal, travel, clinical and test details and results.

The Regional Directors were able to down the line list of the patients and consequently, inform them about the outcome of their test

**Figure 7:**
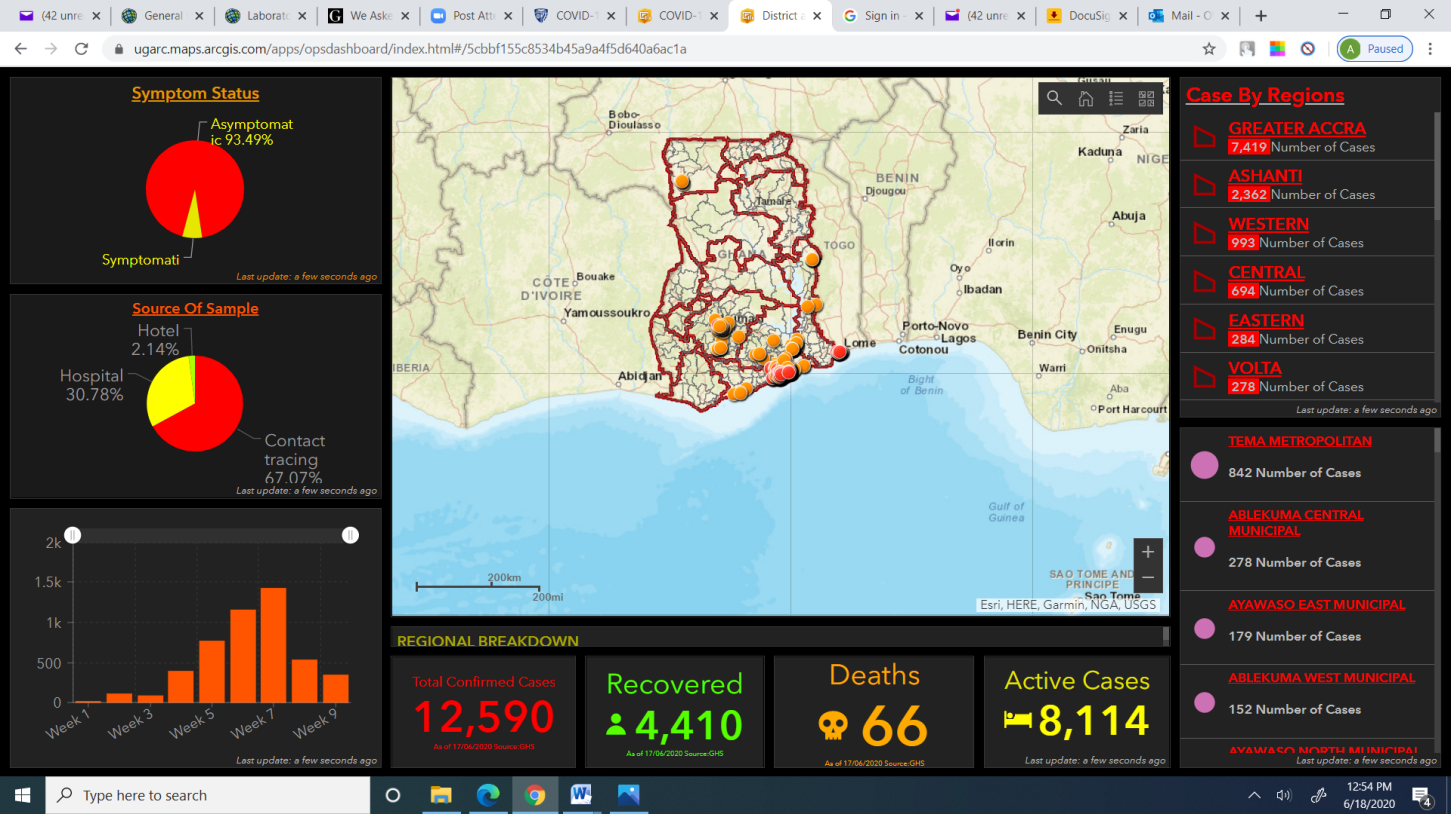
Regional Dashboard with analytics

On the Dashboard, Figure 7 was the main map area which displayed the various locations of all cases recorded in real-time after input from the lab. This symptoms status section displayed the analysed percentage of symptomatic and asymptomatic cases recorded, the source of sample section which dealt with the source of sample through which the case was tested as positive, that is, whether the sample was from contact tracing, hospital or travelers who were placed under 14 days quarantine on arriving in Ghana. The Dashboard provided charts and graphs for more visual effects and display including, daily progression, regional breakdown, deaths, recovered and active case

### Map visualisation of integrated multiple geospatial application's output

**Figure 8:**
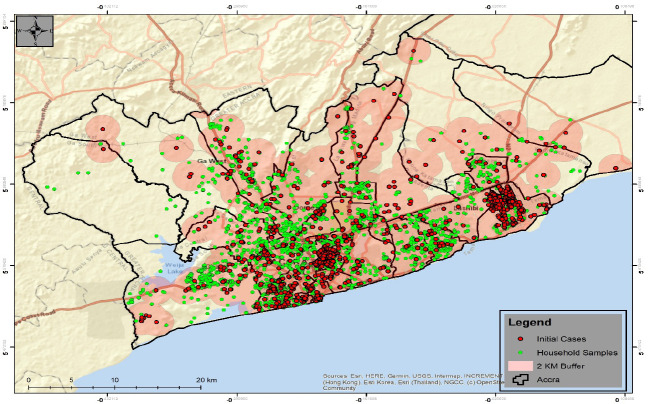
Two km buffer of initial COVID-19 cases and sample points during enhanced testing

**Figure 9:**
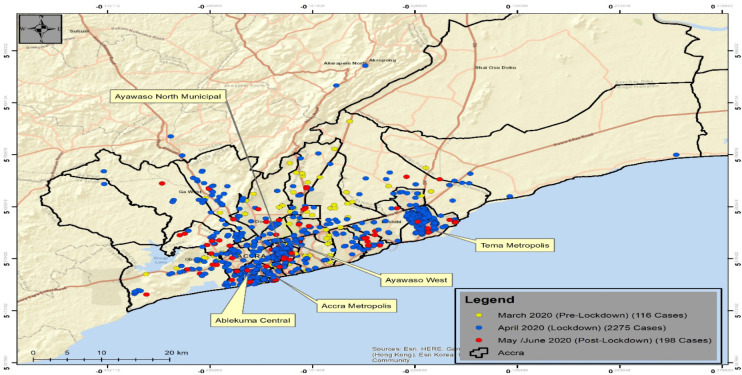
Spot map of COVID-19 cases from pre-lockdown to post lockdown period

The integration of multiple geospatial applications resulted in mapping and visualisation of sample collection points and COVID 19 cases during the enhanced contact tracing when the partial lockdown was imposed on Greater Accra and Kumasi Metropolitan areas. Figure 8 shows locations of initial COVID-19 cases denoted by the red dot with 2km buffer around each. With the enhanced contact tracing, field workers were instructed to collect samples of household members within the 2km of the case. The green dot denotes samples collected for testing during the enhanced contact tracing (Figure 9). The green colour of the sample changes to blue when a sample is confirmed positive see Figure 9. The yellow symbol in the legend shows the geographic locations of the COVID-19 cases before the lockdown. Close to 120 cases were mapped in some districts in Greater for the enhanced contact tracing. The blue symbols denote cases found from the samples collected during the enhanced contact tracing. By the end of April more than 2000 cases were found from the samples collected. These were samples collected from the households tested and were found to be positive. The red symbol denotes post lockdown cases when the enhanced contact tracing eased. Fewer cases were found compared to the lockdown period.

## Discussion

Using various measures to help contain the spread of COVID-19 in the at-risk zones or communities requires an approach to document the real-time location of the cases. This work describes the use of integrated geospatial applications in responding to COVID-19 in Ghana.

During the COVID-19 pandemic, geographical information systems (GIS) were deployed in various settings in responding to the pandemic.^20^ The deployment of integrated GIS applications in the Ghanaian context played a major role in identifying the hotspots of the infection, which helped the country prioritise its response strategies for early detection and control of the infection.

Given the nature of COVID-19 and its way of spread, location information plays a critical role in the response. The collector for ArcGIS was customised to support the location information of COVID-19 cases in Ghana. This aided decision-makers to know which locations or communities were vulnerable to the disease, which neighbourhoods needed more contact tracers, and which ones were newly infected. In other settings, GIS has been used to collect data for decision making in different areas, even in outbreaks and COVID-19.^20^–^25^

An advantage of using the ArcGIS application is the data repository available as the ArcGIS Online. This made the real-time visualisation of data submitted with the ArcGIS collector possible. Real-time visualisation of the GIS data is very important in decision-making and managing human lives in different countries.^24^,^26^,^27^

Data management remains a challenge, especially in a pandemic situation where the number of cases continues to rise with time. It was difficult filling out hard copy case-based investigation forms completely for each sample taken on the field. Pertinent information was therefore lost. With the introduction of a customised application based on Survey 123 for ArcGIS, case-based investigation forms were completed electronically. Samples taken from the field were labelled and scanned with barcodes into the online application system. Relatively, this reduced the length of time used in completing the forms manually. The possibility of missing or rain destroying case-based forms were completely avoided. This application enhanced the receiving and validation of samples by the testing laboratories. This approach was reported to be an accurate, timely and convenient field data collection during emergencies because it ensures prompt management and quick response.^28^

To effectively respond to an outbreak, there is the need to have a system that interconnects the field, laboratory, health directorates and policymakers. This ensures that all stakeholders have the right information communicated to them on time for accurate decisions. The Regional Supervisors backend web applications created allowed patient results to be assessed by the regional health directorates. Timely results released to the directorate aided in identifying positives in the shortest possible time and communicating results to patients. This was because the application provided real-time tracking location and status of tested samples. This procedure was important in decision-making and improved data transparency and assisted in information dissemination.^29^

Again, the interactive dashboards for policymakers combined spatial analytics capabilities with time filters to view test results on country-wide, regional and district bases. This facilitated easy understanding of results by the Government and other decision-makers. The use of real-time data and coordination of information enabled policymakers to adjust management strategies in near real-time within the COVID-19 hotspot zones identified in the country.

The application improved the communication of test results in terms of accuracy and timeliness. The testing labs scan using barcode readers and enter the details of the individual test results with many pre-coded options. Time for typing results improved, the accuracy of results communicated improved while regional directors and Decision Makers Dashboard automatically updated instantly with the entered lab results. This allows regional Directors to access test results of individuals patients, including their location, as soon as the laboratories enter the results. The results show that communication through map-based dashboards offers accessible information.^29^

The deployment of this integrated system helped map and identify case locations, contact tracing locations, and the characteristics of neighbourhoods and communities where the cases were being reported. Based on this application, it was observed that all the initial cases were located in Greater Accra Metropolitan Area (GAMA) and Greater Kumasi Metropolitan Area (GKMA). Again, it was also observed that all the initial cases were in high income residential areas of Accra Metro and Ayawaso West Wogon. The objective of the enhanced contact tracing during the lockdown was to identify all possible cases through contact with initial cases, isolate and treat them to reduce the spread of the COVID-19 disease. The map visualisation of integrated multiple geospatial application output (Figure 9) shows that many cases that otherwise would have been left in the communities to infect other people have been identified and isolated or treated. Hence, the lockdown and enhance contact tracing used by Ghana have largely achieved their objectives.

Some limitations encountered during the study were the phased approach used in implementation of the various applications. For each application, contact tracers and health workers needed to be trained on how to be used. Due to the little time they had on their hands, brief training sessions were held for them. This led to initial results with some lapses and gaps such as inability to sync the data properly, inaccurate geospatial coordinates and difficulty in completing some of the fields on the case-based form. Supervisors were assigned to each district to provide supportive supervision and any technical assistance needed to solve this problem. Again, all issues seen with the data was relayed to supervisors who followed upon them to be correctly captured.

Implementing the lockdown with extreme measures of restriction in mobility, social distancing, closure of schools, banning of mass gathering, isolation and quarantine helped undertake the enhanced contact tracing in the various communities. During the period (Figures 9 &10), more cases were detected, isolated and treated; hence, the aim of the lockdown was large achieved^31,32^

## Conclusion

This study describes how the integration of multiple geospatial applications with spatial intelligence capabilities effectively responded to COVID-19 in Ghana during the early stages of the pandemic (from March to the end of April 2020). Specifically, the study has demonstrated how the integration of geospatial applications was used for effective communication between contact tracers, field sample collectors, testing laboratories, Regional Health directors and Government Decision Makers. This work demonstrates that an integrated geospatial system can create a network between the field, laboratory, health workers and decision-makers to effectively respond to an outbreak.

## Figures and Tables

**Figure 5:**
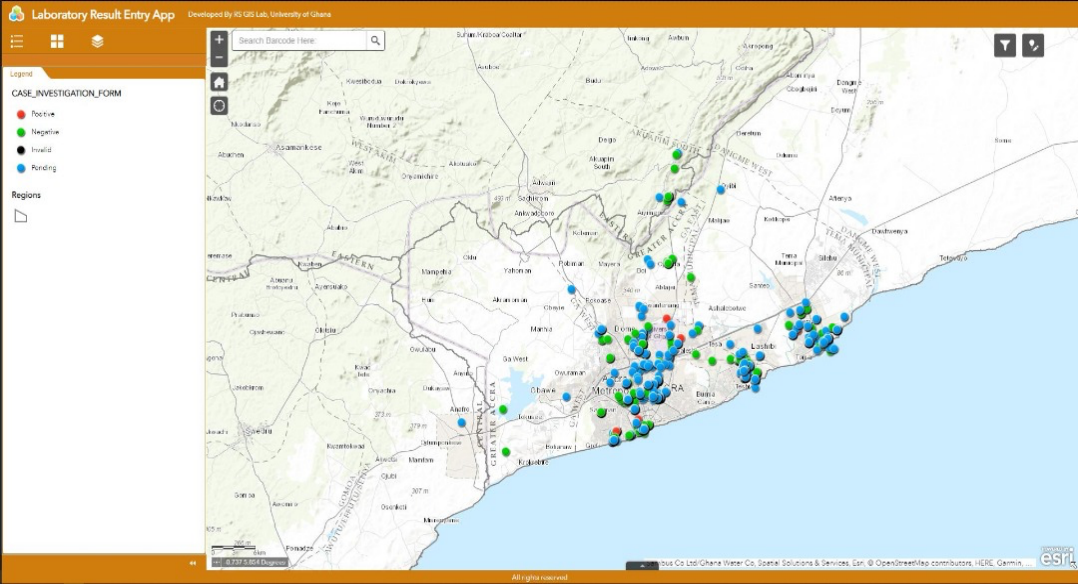
User interface of the lab backend web application
